# Inter-Examiner and Inter-Instrument Agreement of the Myopia Master with the IOL Master 700 and NVisionK 5001 in Myopic Children †

**DOI:** 10.3390/children12020121

**Published:** 2025-01-23

**Authors:** Emma L. McConnell, Cameron Woods, Lesley Doyle, Jane M. Fulton, Kathryn J. Saunders, Sara J. McCullough

**Affiliations:** 1 Centre for Optometry and Vision Science, Ulster University, Coleraine BT52 1 SA, UK; woods-c29@ulster.ac.uk (C.W.); la.doyle@ulster.ac.uk (L.D.); j.fulton@ulster.ac.uk (J.M.F.); kj.saunders@ulster.ac.uk (K.J.S.); sj.mccullough@ulster.ac.uk (S.J.M.); 2 Northern Ireland Clinical Research Network, Belfast Health and Social Care Trust, Royal Victoria Hospital, Belfast BT12 6BA, UK; 3 Northern Ireland Clinical Research Facility, Queen’s University Belfast, Belfast BT9 7AB, UK

**Keywords:** myopia, inter-instrument agreement, myopia master, childhood myopia, inter-examiner agreement, ocular biometer, myopia management, axial length, spherical equivalent refraction, corneal curvature

## Abstract

**Background/Objectives**: The measurement of axial length (AL) is important to monitor the success of interventions to slow childhood myopia, but traditional biometers are expensive for clinicians to acquire. To address this, Oculus has developed the Myopia Master (MM), which measures auto-refraction (AR), corneal curvature (CC) and AL. This study compared inter-instrument and inter-examiner measures on the MM, IOL Master 700 (Carl Zeiss Meditec, Jena, Germany) and NVisionK-5001 (Shin-Nippon, Tokyo, Japan) in myopic children. **Methods**: Fifty-six myopic children participated (mean age 11.93 ± 1.93 years). A first examiner obtained the following under cycloplegia: (i) AR using the NVisionK, (ii) AL and CC using the IOL Master 700 and (iii) AR, AL and CC using the MM. The latter measures were repeated by a second examiner to assess inter-examiner agreement of the MM. Mean differences (MD) and 95% limits of agreement (LoA) were calculated. Spherical equivalent refraction (SER) was calculated using AR values. Acceptable LoA for AL, SER and CC were defined as ±0.05 mm, ±0.40 D and ±0.06 mm, respectively. **Results**: Inter-instrument LoA between the MM and IOL Master 700 for AL and CC were acceptable (MD 0.02 mm; 95%LoA −0.02 to 0.06 mm and MD 0.025 mm, 95% LoA −0.03 to 0.08 mm, respectively). SER measures between the NVisionK-5001 and MM were not acceptable (MD −0.44 D, 95% LoA −0.91 to 0.03 D). SER values were, on average, 0.44 D more negative when using the MM. Inter-examiner measures on the MM were all acceptable (AL MD 0.00 mm; 95% LoA −0.04 to 0.04 mm; SER MD −0.01 D; 95% LoA −0.33 to 0.32 D; CC MD 0.01 mm; 95% LoA −0.03 to 0.05 mm). **Conclusions**: The MM demonstrated excellent inter-examiner repeatability. Inter-instrument agreement between the MM and IOL Master 700 for the AL and CC measures showed excellent agreement. The MM over-estimated myopia SER compared with the NVisionK-5001.

## 1. Introduction

It is widely reported that the prevalence of myopia is increasing worldwide [1,2,3]. Considering this, interventions to slow the progression of myopia in children are now readily available in the form of optical, pharmacological and light therapy interventions. The efficacy of these treatments is summarised in the most recent Cochrane living systematic review and meta-analysis on myopia interventions [4]. There now exists a strong evidence base to support the provision of myopic interventions to children in an attempt to slow progression such that the World Council of Optometry (WCO) now states that “simply correcting the refractive error [of a myopic child] is no longer sufficient, and myopia management should not be optional and rather be an obligation of optometrists” [5]. This strong recommendation from the WCO, coupled with the increasing commercial availability of myopia management interventions, has prompted optometrists to offer myopia management to their paediatric myopic patients in clinical practice.

Axial elongation is the primary concern with regard to myopic progression due to the increased risk of developing pathological complications in later life caused by excessive stretching of the eye [6,7]; therefore, it is recommended that practitioners monitor axial growth to determine the efficacy of myopia management rather than solely relying on the change (or lack thereof) in magnitude of refractive error. In addition, axial length measurements are more precise and repeatable than refractive error measurements. The inclusion of traditional biometers, such as the IOL Master (Carl Zeiss Meditex, Jena, Germany) or Lenstar (Haag-Streit, Switzerland), within a high street practice presents several barriers, including the cost and space required to house an additional instrument. The Myopia Master (Oculus, Wetzlar, Germany) provides an alternative solution as an all-in-one device which measures refractive error, axial length and corneal curvature, negating the need for separate instruments to acquire these measurements. Furthermore, the Myopia Master offers additional features, including the ability for practitioners to compare individual patients’ data to normative eye growth charts, apply risk factor questionnaires, present treatment recommendations, produce customised take-home reports for parents and conduct a follow-up analysis at a child’s subsequent clinic visits.

The International Myopia Institute (IMI) has published recommendations regarding the instrumentation researchers should use when conducting interventional myopia clinical trials. For the measurement of cycloplegic refractive error, an open-field auto-refractor is recommended to reduce the variability caused by residual accommodation and instrument myopia [8]. For axial length, the use of non-contact optical biometers is recommended, as they provide high-precision measures compared to contact ultrasound biometry, which produces greater measurement variability [8]. Studies now exist that compare axial length and corneal curvature measurements obtained on the Myopia Master with the ‘gold standard’ IOL Master 700 in both adult and paediatric populations, with conclusions varying as to whether these devices can be used interchangeably (discussed in detail later). Few studies have examined the agreement of the Myopia Master with commonly used open-field autorefractors, which have traditionally been used to inform research evidence and, consequently, recommendations for evidence-based clinical practice. Pederson et al. (2023) compared autorefraction results on the Myopia Master to those obtained on the Huvitz HRK-8000A and found that measures obtained were not within acceptable limits of agreement and thus should not be used interchangeably [9]. Their study included an adult population but reported that data are currently lacking for paediatric populations, the demographic for which the Myopia Master was predominantly designed. This study aims to evaluate the agreement and inter-examiner reproducibility of biometric and refractive error measurements taken under cycloplegia by the Myopia Master compared to the IOL Master 700 and Shin-Nippon NVisionK 5001 open-field autorefractor in myopic children. If agreement between these traditional instruments and the Myopia Master exists, practitioners can have confidence that measurements obtained using this device are accurate, reliable and translate well with the research evidence, potentially enabling the incorporation of this convenient device into future research trial protocols.

## 2. Materials and Methods

### 2.1. Participants

Children who were taking part in myopia research trials at Ulster University between May and December 2022 were invited to participate. Measurements were taken on the Myopia Master (Oculus, Wetzlar, Germany) when they attended for scheduled trial visits. Written informed parental consent and written participant assent were obtained prior, over and above those already obtained for trial participation prior to conducting study procedures. The study was approved by the Ulster University Research Ethics Committee and adhered to the tenets of the Declaration of Helsinki.

### 2.2. Study Instrumentation

#### 2.2.1. Oculus Myopia Master

To measure axial length, the Myopia Master uses non-contact partial coherence interferometry with an 880 nm light source. Six measurements are acquired in automatic succession. All successful individual measures are displayed with their corresponding signal-to-noise ratio, alongside the mean of all successful measures. Corneal curvature measures are determined through reflected test spots projected onto the central 15 degrees of the cornea (approx. central 3–4 mm of the cornea) captured by a digital camera sensor. Autorefraction measures are obtained by an infrared light source (880 nm) directed through the pupil centre onto the retina, from which it is reflected back into the instrument. Refractive error components (sphere, cylinder and axis) are determined according to the deviation of reflected light from the instrument shutter location.

#### 2.2.2. Zeiss IOL Master 700 and Shin-Nippon NVisionK 5001

The Zeiss IOL Master 700 (Carl Zeiss Meditec, Jena, Germany) [10] and Shin-Nippon NVisionK (Shin-Nippon, Tokyo, Japan) [11] have been described previously.

In brief, the IOL Master 700 is a non-invasive ocular biometer that uses swept-source ocular coherence tomography (SS-OCT) to acquire measures of axial length, central corneal thickness, anterior chamber depth and lens thickness. The use of SS-OCT technology facilitates faster scanning acquisition time. Multiple measurements are captured automatically, with individual and average values available for assessment post-acquisition.

The NVisionK 5001 (also branded as the Grand Seiko WR-5100K) is an open-field autorefractor that acquires autorefraction and keratometry values. The device images a ring target of infrared light reflected from the retina. A lens is moved on a motorised track to place the ring target in focus. This image is then analysed digitally to calculate refractive error. The open-field design relies on fixation on an external target located at a distance in front of the patient, hence reducing the risk of proximal accommodation [11,12].

### 2.3. Examination Procedures

Following informed consent, demographic information was obtained from each child (age, gender, ethnicity). The monocular best-corrected distance visual acuity was obtained using the Early Treatment of Diabetic Retinopathy Study (ETDRS) letter chart. Cycloplegia was achieved in each eye via two methods dependent on the study protocol of the trial in which the child was originally enrolled (either two drops of tropicamide 1% instilled five minutes apart or one drop of proxymetacaine hydrochloride 0.5% followed by one drop of cyclopentolate hydrochloride 1%). Biometric and refractive measurements were obtained at least 30 min following instillation of the second eye drop.

The following measurements were obtained from both eyes of each participant:

1. Refractive error measured using the NVisionK 5001 by a first examiner.
2. Axial length and corneal curvature measured using the IOL Master 700 by a first examiner.
3. Refractive error, axial length and corneal curvature measured using the Myopia Master by a first examiner.
4. Refractive error, axial length and corneal curvature measured using the Myopia Master by a second examiner.

Measurements were obtained by four examiners across the study duration (C.W., E.L.M., J.M.F., S.J.M.). For each instrument, the participant was instructed to place their forehead and chin against the respective rests and asked to focus their gaze on a fixation target; the IOL Master and Myopia Master fixation targets were internal, whereas the fixation target for the open-field autorefractor was a Maltese cross situated on a wall at a distance of 2 m directly in front of the participant. Calibration of the instruments was checked at the beginning of each assessment day prior to obtaining measurements.

### 2.4. Statistical Analysis

Statistical analysis was conducted using SPSS Statistics software version 28. For each participant, the mean axial length and corneal curvature were calculated from measurements acquired on each instrument. A minimum of two measures were used for axial length and three for corneal curvature. Spherical equivalent refraction (SER) was calculated for autorefraction measures obtained on both instruments by adding half of the cylindrical value to the sphere for a 0 mm vertex distance. For corneal curvature, a mean of both radii of curvature was used in subsequent analyses. For SER, five measurements on the NVisionK 5001 and three on the Myopia Master were taken, with the calculated representative value taken for analysis. Bland–Altman analysis was used to determine the agreement and inter-examiner reproducibility between the instruments. The distribution of data was assessed using the Shapiro–Wilk test for normality, with a *p*-value > 0.05 indicating normally distributed data. Mean difference (MD), standard deviation (SD) and 95% limits of agreement (LoA) were calculated from measures obtained by the different instruments for axial length and refractive error. The same metrics were calculated from measures obtained by each examiner on the Myopia Master to determine the inter-examiner reproducibility of the instrument. Refraction measurements obtained on the Myopia Master were graded for quality on a scale of 1–9; all measurements included in the analysis were of a quality of at least 8, as this is defined as ‘good’ quality [13]. Bland–Altman plots [14] were constructed to describe both the reproducibility of the Myopia Master (comparison of measurements obtained by two examiners) and the agreement between the IOL Master 700 and NVisionK 5001 with the Myopia Master. Acceptable limits of agreement for axial length, SER and corneal curvature were defined as ±0.05 mm, ±0.40 D and ±0.06 mm, respectively, as described by Pederson et al. (also derived from Brennan et al., 2021) [9,15]. Only data from the right eye are presented, as measures obtained from the right and left eyes were highly correlated (Pearson’s correlation co-efficient r > 0.886, *p* < 0.001 for all measures). Pearson’s correlation (or Spearman’s correlation for non-normally distributed data) was used to determine the presence of proportional bias between the mean and mean difference values for each measure. A *p*-value of <0.05 was considered statistically significant, indicating proportional bias in the measures.

A previous study investigating inter-instrument agreement between two optical biometers applied to children was used to determine sample size [16]. Using recommended sample size calculations for agreement studies [17], an estimated sample size of 56 participants was required and achieved based on a standard deviation of 0.022 mm (derived from Leighton et al., 2022 [16]) and desired confidence interval of 0.01 mm (i.e., the smallest measurable step on the biometers under testing).

## 3. Results

### 3.1. Population

Fifty-six myopic children participated in the study, ranging in age from 7.75 to 15.75 years old (mean age 11.93 ± 1.93 years; 50% male). The mean ± SD SER was −2.86 ± 1.29 D (range −0.13 to −6.00 D; values based on objective cycloplegic autorefraction measures obtained using the NVisionK 5001 (the SER for one child was −0.13 D using this device; however, their current spectacles and autorefraction results on the Myopia Master were <−1.00 D, which is why they are included in this sample)). The majority of children were white (92.9%, *n* = 51), three were Chinese (5.4%) and one was mixed white/Asian (1.8%).

### 3.2. Inter-Examiner Reproducibility of the Myopia Master

**Figure 1:**
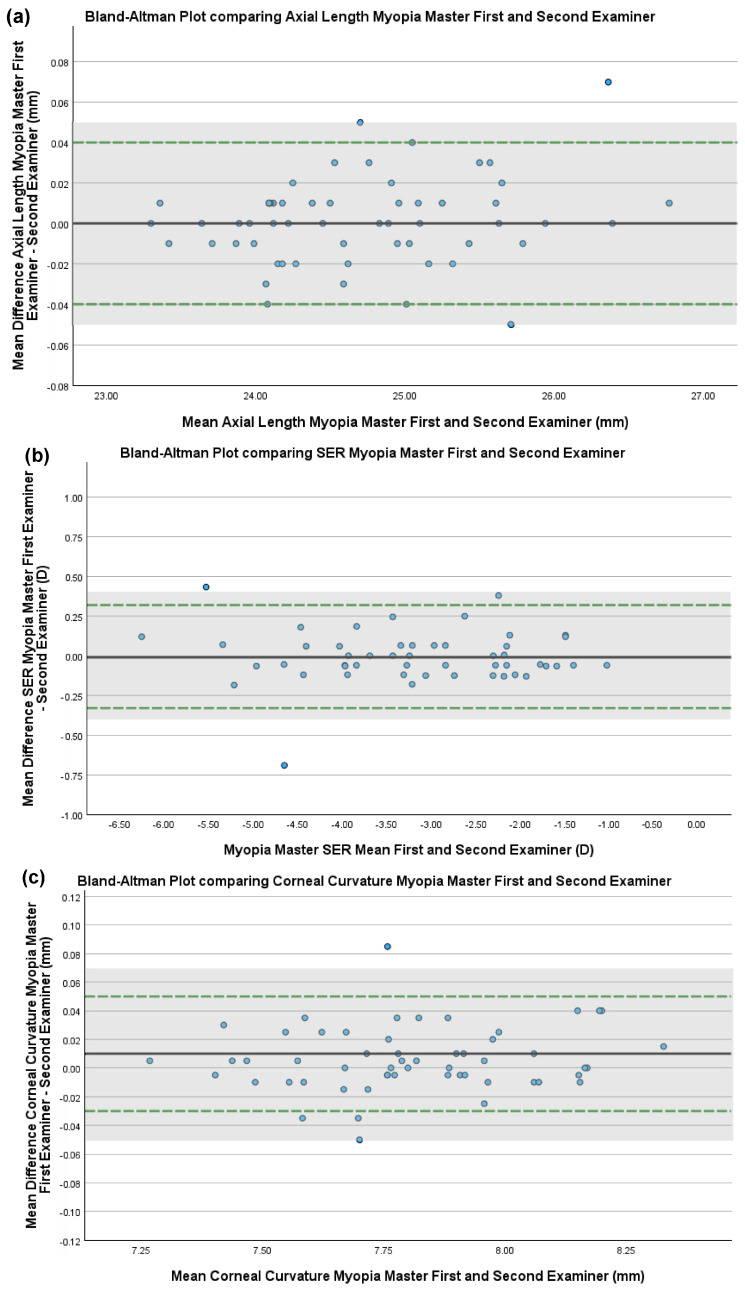
Bland–Altman plots showing the inter-examiner reproducibility/level of agreement of the Myopia Master for (**a**) axial length, (**b**) spherical equivalent refraction (SER) and (**c**) corneal curvature. The mean difference is illustrated by a solid black line, and the upper and lower 95% limits of agreement are illustrated by dashed green lines. Blue dots indicate individual data points. Shaded grey boxes indicate pre-defined acceptable limits of agreement (±0.05 mm for axial length, ±0.40 D for SER and ±0.06 mm for corneal curvature [9,15]).

The mean (±SD) and range of axial length, SER and corneal curvature measures obtained by a first and second examiner using the Myopia Master are shown in Table 1. Six SER measures from the Myopia Master and one corneal curvature measure did not meet the quality standards for inclusion. Mean difference, SD and 95% LoA are shown in Table 2. Figure 1a–c show Bland–Altman plots for the inter-examiner reproducibility for axial length, SER and corneal curvature measures obtained on the Myopia Master. Using pre-defined acceptable limits of agreement (±0.05 mm, ±0.40 D and ±0.06 mm for axial length, SER and corneal curvature, respectively) [9,15], all measures obtained on the Myopia Master by two separate examiners were within acceptable limits of agreement (Table 2).

### 3.3. Inter-Instrument Agreement

#### 3.3.1. Myopia Master and IOL Master 700

**Figure 2:**
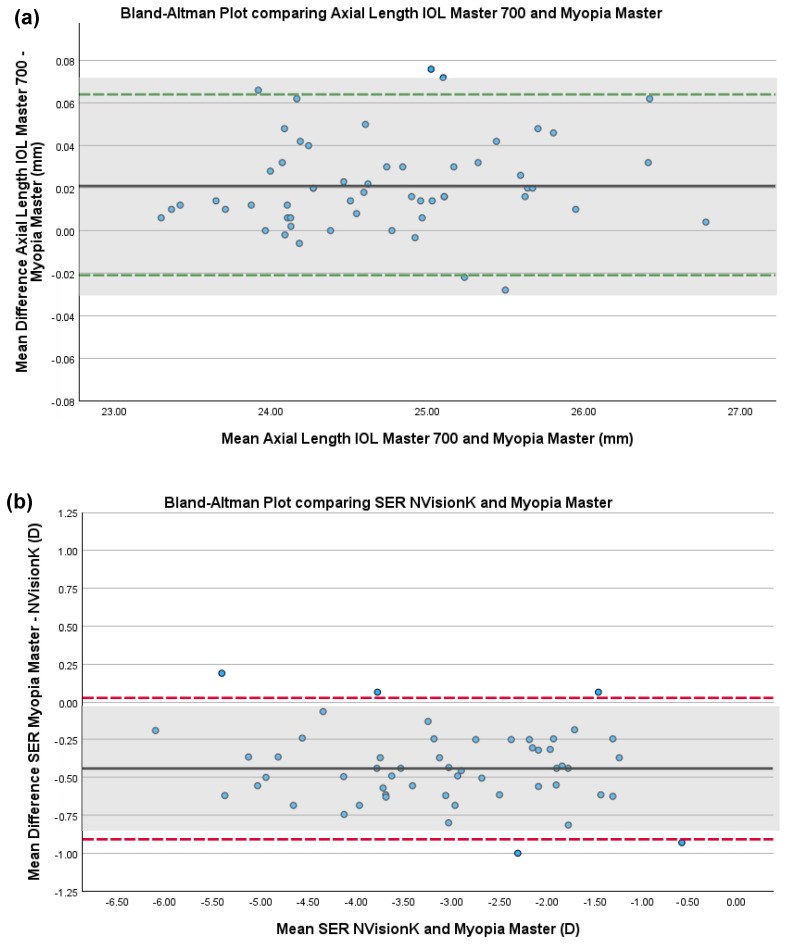

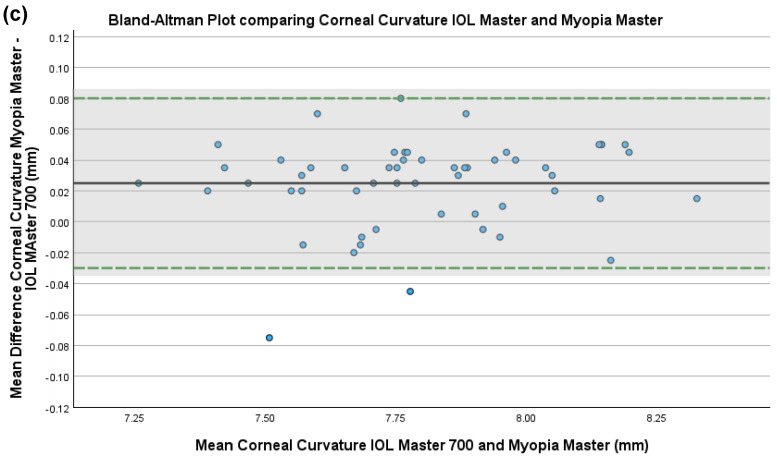
Bland–Altman plots showing the level of agreement showing the inter-instrument level of agreement for (**a**) axial length between the IOL Master 700 and Myopia Master, (**b**) spherical equivalent refraction (SER) between the Myopia Master and NVisionK 5001 and (**c**) corneal curvature between the IOL Master 700 and Myopia Master. Mean difference is illustrated by a solid black line and upper and lower 95% limits of agreement illustrated by dashed lines. Green dashed lines represent measurements are within acceptable limits of agreement, whereas red dashed lines fall outside acceptable limits of agreement. Blue dots indicate individual data points. Shaded grey boxes indicate pre-defined acceptable limits of agreement (±0.05 mm for axial length, ±0.40 D for SER and ±0.06 mm for corneal curvature [9,15]).

The mean (±SD) and range of axial length and corneal curvature measures obtained using the Myopia Master and IOL Master 700 are shown in Table 1. One corneal curvature measure obtained on the Myopia Master did not meet the quality standards for inclusion; all other measures were included. Mean difference, SD and 95% LoA are shown in Table 2. Figure 2a–c show Bland–Altman plots for the inter-instrument agreement for axial length and corneal curvature, respectively. Using pre-defined acceptable limits of agreement (±0.05 mm and ±0.06 mm for axial length and corneal curvature, respectively) [9,15], all measures obtained on the Myopia Master and IOL Master 700 were within acceptable limits of agreement (Table 2).

#### 3.3.2. Myopia Master and NVisionK 5001

The mean (±SD) and range of SER measures obtained using the Myopia Master and NVisionK 5001 are shown in Table 1. Two SER measures obtained on the Myopia Master did not meet the quality standards for inclusion; all other measures were included. Mean difference, SD and 95% LoA are shown in Table 2. Figure 2b shows a Bland–Altman plot for the inter-instrument agreement. Using pre-defined acceptable limits of agreement (±0.40 D) [9,15], measures obtained on the Myopia Master and the NVisionK 5001 were not within acceptable limits of agreement (Table 2). Measures obtained on the Myopia Master were on average 0.44 D more negative than those obtained on the NVisionK 5001. Measurements from 87.5% of participants were within the acceptable LoA.

Pearson’s/Spearman’s correlation showed no proportional bias for all measures of spherical equivalent refraction, axial length or corneal curvature acquired on all instruments (*p* > 0.127 for all).

## 4. Discussion

With the increased uptake of myopia management in clinical practice, it is imperative that practitioners have access to accurate and reliable instrumentation to monitor the onset and progression of myopia in paediatric patients, and to assess whether a myopia management intervention is having the desired effect of slowing eye growth. Several instruments designed for use in clinical myopia management are now commercially available (e.g., Oculus Myopia Master, Topcon MYAH, Lenstar Myopia). The aim of this study was to determine, in a population of paediatric myopes, whether spherical equivalent refraction (SER), axial length and corneal curvature measures obtained using the Myopia Master were in agreement with measures obtained using gold standard instrumentation (IOL Master 700 and NVisionK 5001) that has been widely used for monitoring the progression of myopia in a research setting.

The present study shows that axial length and corneal curvature measurements acquired on the Myopia Master and the IOL Master 700 were within pre-defined acceptable limits of agreement (±0.05 mm and ±0.06 mm, respectively), indicating that the Myopia Master produces accurate results comparable to gold standard instrumentation. A change in axial length of 0.10 mm is approximately equivalent to a 0.25 D change in spherical equivalent refraction [15,18,19]. The mean difference between the IOL Master 700 and Myopia Master in the present study was 0.02 mm, signifying a clinically insignificant difference between the two instruments. This finding has important implications for clinical practice: the Myopia Master is an all-in-one device, which is advantageous for practitioners who may have limited space available to acquire multiple instruments for ocular biometry and autorefraction. The results of the present study provide practitioners with confidence that axial length and corneal curvature measurements made with the Myopia Master are as accurate and reliable as those obtained with standard research equipment. Furthermore, the children included in the present study had a range of axial lengths, from 23.30 mm to 26.78 mm. No proportional bias was evident in the measures, indicating that the Myopia Master’s accuracy is not influenced by axial length.

In contrast, the refractive error measures obtained using the Myopia Master and NVisionK 5001 were not within accepted limits of agreement (±0.40 D), with the Myopia Master producing, on average, more negative results (mean −0.44 D) than the NVisionK 5001. This is not an uncommon finding when comparing autorefraction devices. Pederson et al. (2023) compared the open-field Huvitz HRK-8000A with the Myopia Master and reported that their results were, on average, −0.19 D more negative on the Myopia Master [9]. A possible explanation for this is due to the closed-field design of the Myopia Master, which may induce a temporary increase in accommodation, referred to as proximal accommodation or instrument myopia, a well-established phenomenon observed when viewing through an optical instrument [20]. Under cycloplegia, the potential for this proximal/instrument myopia effect should be minimal. Children in the present study were cyclopleged with either tropicamide 1% or cycloplentolate 1%, depending on the protocol of the myopia trial they were originally enrolled in. However, the mean differences between the NVisionK and Myopia Master did not differ significantly when comparing the two cycloplegic agents (mean −0.4407 + 0.22 for the tropicamide 1% group vs. −0.4403 + 0.25 for the cyclopentolate 1% group, t = −0.006, *p* = 0.995), indicating that the cycloplegic agents used did not affect the SER results obtained. Ye et al. (2022) compared spherical equivalent refraction results obtained on the Myopia Master and the Nidek ARK-1 in ametropic children (hyperopes, myopes and astigmats) [21]. The mean difference between instruments was 0.40 D (95% LoA −0.26 to 1.16 D). Similar to the results of the present study and those reported by Pedersen et al. (2023), the Myopia Master produced more negative results. Both the Myopia Master and Nidek ARK-1 instruments are closed-field in design, which should make any proximal accommodation differences minimal in these measures. It has previously been reported that the Nidek ARK-1 produces more positive results compared to subjective refraction [22]. This positive bias may account for the difference between the ARK-1 and Myopia Master in Ye et al.’s population.

Despite the inter-instrument difference, inter-examiner measures on the Myopia Master were within acceptable limits of agreement (−0.33 to 0.32 D). Similarly, inter-examiner measurements of axial length and corneal curvature acquired on the Myopia Master were within acceptable limits of agreement (−0.04 to 0.04 mm and −0.03 and 0.05 mm respectively). These findings imply that the Myopia Master produces consistent results such that practitioners can have confidence in using the instrument to monitor refractive change over time in individual patients.

Several studies have compared measurements obtained from the Myopia Master with those obtained from the IOL Master 700 across a variety of populations. The results are summarised in Table 3. In keeping with the results of the present study, Garcia Ardoy et al. (2023) and Ye et al. (2022) report that, in their population, axial length measures obtained on the Myopia Master were on average 0.02 mm shorter than measures obtained on the IOL Master 700 [21,23]. In contrast, however, the LoA between instruments reported in both studies exceeded acceptable values (−0.101 to 0.17 4 mm and −0.07 to 0.12 mm, respectively; Table 3). This is at odds with the outcomes in the present study (−0.02 to 0.06 mm). The children in the present study were enrolled in ongoing myopia clinical trials. As part of the trials, they were required to attend the clinic at regular intervals for repeat measures of cycloplegic autorefraction and axial length. As such, these children were familiar with the instruments included in the present study, meaning that their cooperation was typically good compared to children who have no previous experience in their use. This may account for the differences noted between our study and those of previous studies. Furthermore, the population included in Ye et al. (2022) was, on average, younger than those included in the present study [21]. Younger children may find it difficult to maintain good positioning and fixation during measurements, leading to greater variability in results. Hessler et al. (2023) also reported that axial length measures from the Myopia Master were 0.02 mm shorter than those from the IOL Master 700 in their adult population [24]. They reported narrower LoA (−0.06 to 0.01 mm), which may be due to the adult population yielding more stable fixation, similar to children who are more familiar with the requirements of the instrumentation. Use of cycloplegia is another possible explanation for the differences in LoA noted between the present study and previous studies. Measurements by Garcia Ardoy et al. (2023) were captured prior to instilling cycloplegic eye drops, whereas our measurements were obtained following cycloplegia. Although clinically insignificant, small differences in axial length measurements obtained pre- and post-cycloplegia have been observed in children using the IOL Master [25,26] and Myopia Master [27], which may account for the differences in LoA between the present study and those of Garcia Ardoy et al. (2023).

Ortiz-Toquero et al. (2024) conducted a study on inter-examiner reproducibility measures of axial length and corneal curvature on a group of adult myopes (mean age 24.7 ± 5.8 years) using the Myopia Master [28]. The mean difference in axial length measures between examiners was 0.00 ± 0.02 mm, with 95% LoA from −0.05 to 0.05 mm, values that are in agreement with the findings of the current study. The authors analysed corneal curvature meridians separately, rather than as a combined mean. For both the flat and steep meridian, the mean difference in measures obtained by two separate examiners was 0.00 ± 0.01 mm, and the 95% LoA for the flat and steep meridians were −0.03 to 0.03 mm and −0.02 to 0.03 mm, respectively. The results of the current study report similar findings, which further indicates that the measures obtained from the Myopia Master by two different examiners were consistent and reliable in both children and adults.

In contrast to the results of the present study, where measures made by the Myopia Master and IOL Master 700 concur with pre-defined acceptable limits of agreement, comparisons with other ocular biometers may not provide the same results. Chamarty and Verkicharla (2023) compared axial length and corneal curvature values obtained on the Myopia Master and Lenstar 900 and reported that results between the two instruments were not within acceptable limits of agreement in both paediatric and adult populations [29]. Similarly, Otriz-Toquero et al. (2024) reported that caution should be used when considering using corneal curvature values obtained using the Topcon MYAH (another device developed specifically for myopia management in clinical practice) interchangeably with the Myopia Master. However, axial length values recorded with the two instruments were within acceptable limits of agreement and, therefore, could be used interchangeably [28]. Considering the differing conclusions between studies on whether measurements obtained on different instruments can be used interchangeably, it is important that practitioners and researchers interpret study results considering the context of the instrumentation used, as well as participant characteristics such as refractive error, age and ethnicity.

**Table 3:** Comparison of studies that compared axial length and corneal curvature measurements acquired on the Myopia Master and IOL Master 700. SD = standard deviation, MD = mean deviation, LoA = limits of agreement. Orthok = orthokeratology, mm = millimetres.

Authors (Year)	Subjects (n)	Cycloplegia	Mean Age (SD) (Years)	Instruments Compared	Refractive Errors	Axial Length MD (SD) (mm)	Axial Length 95% LoA (mm)	Corneal Curvature MD (SD) (mm)	Corneal Curvature 95% LoA (mm)
Present study	Children (56)	Yes	11.93 (1.93)	Myopia Master v IOL Master 700	Myopes	0.02 (0.02)	−0.02 to 0.06	0.025 (0.03)	−0.03 to 0.08
Pederson et al. (2023) [9]	Adults (74)	Yes	22.8 (3.7) (range 19–41 years)	Myopia Master v IOL Master 700	52 myopic, 32 emmetropic, 55 hyperopic	−0.004 (0.047)	−0.097 to 0.089	0.035 (0.028)	−0.02 to 0.09
Ye et al. (2022) [21]	Children (125)	Yes	Males 8.27 (2.68); females 8.31 (2.20) (range 3–15 years)	Myopia Master v IOL Master 700	Myopia, hyperopia or astigmatism (n in each group not provided)	0.02	−0.07 to 0.12	0.02	−0.06 to 0.12
Mattern et al. (2023) [30]	Children (22); 16 with repeat data	Not stated	11.28 (2.4)	Myopia Master v IOL Master 700	Myopes	0.01	−0.06 to 0.08	0.07	−0.12 to 0.26
Garcia Ardoy et al. (2023) [23]	Children (120) split into spectacle and Orthok groups	No	Spectacle wearing group 12.3 (2.3) (range 8–16 years); Orthok group 13.4 (2.1) (range 9–16 years)	Myopia Master v IOL Master 700	Myopes	Spectacles group 0.036 (0.07); Orthok group 0.044 (0.097)	Spectacles group −0.101 to 0.174; Orthok group −0.146 to 0.234	Spectacles group 0.05 (0.04); Orthok group 0.05 (0.10)	Spectacles group −0.03 to 0.13 mm Orthok group −0.14 to 0.24
Hessler et al. (2023) [24]	Adults (120)	No	37.7 (15.5) (range 18–72 years)	Myopia Master v IOL Master 700	73% myopes	−0.02 (0.02)	−0.06 to 0.01	−0.07 (0.04)	−0.16 to −0.02

The present study highlights a number of strengths. Primarily, the population included in this study were myopic children aged 7.75 to 15.75 years, the primary group of patients on which the Myopia Master is most likely to be used. Furthermore, the inter-examiner reproducibility of the Myopia Master was assessed, which to our knowledge, has not yet been reported. The potential for inter-operator variation is an important consideration for determining the utility of the Myopia Master in monitoring myopia progression, particularly in primary eye care settings where patients may be assessed by different clinicians at subsequent visits.

A limitation of this study is that we did not include children who were emmetropic or pre-myopic. The Myopia Master, and similar instruments, are important tools for practitioners to utilise when introducing myopia management into their practice. It is important to know how children’s eyes grow and develop prior to the onset of myopia, which allows practitioners to assess whether introducing a myopia management intervention is having the desired effect of slowing refractive and axial progression; therefore, reliability data in pre-myopic children using these instruments are warranted. Furthermore, cycloplegia was used in this study, which may not reflect everyday clinical practice when monitoring myopia in paediatric patients.

When used to assess the axial length of myopic children, the Myopia Master produces reliable and repeatable measures. Refractive error measurements are reproducible but slightly over-estimate the level of myopia compared with measures obtained with the NVisionK 5001.

## Figures and Tables

**Table 1:** Mean ± SD and range of axial length, SER and corneal curvature values for each instrument, as well as between the first and second examiner for the Myopia Master.

Inter-Instrument Agreement Measures:				
**Parameter**	**Myopia Master First Examiner**	**IOL Master 700**		**NVisionK 5001**
**Mean axial length** ± **SD** (mm) (range) (*n* = 56)	24.72 ± 0.79 (23.30 to 26.78)	24.74 ± 0.79 (23.30 to 26.78)		
**Mean SER** ± **SD** (D) (range) (*n* = 54)	−3.30 ± 1.26 (−1.06 to −6.19)			−2.86 ± 1.29 (−0.13 to −6.00)
**Mean corneal curvature** ± **SD** (mm) (range) (*n* = 55)	7.81 ± 0.24 (7.27 to 8.34)	7.79 ± 0.23 (7.25 to 8.32)		
**Inter-examiner reproducibility measures:**				
	**Myopia Master** **First Examiner**		**Myopia Master** **Second Examiner**	
**Mean axial length** ± **SD** (mm) (range) (*n* = 56)	24.72 ± 0.79 (23.30 to 26.78)		24.72 ± 0.78 (23.30 to 26.77)	
**Mean SER** ± **SD** (D) (range) (*n* = 50)	−3.18 ± 1.22 (−1.06 to −6.19)		−3.18 ± 1.22 (−1.00 to −6.31)	
**Mean corneal curvature** ± **SD** (mm) (range) (*n* = 55)	7.81 ± 0.24 (7.27 to 8.34)		7.81 ± 0.24 (7.27 to 8.32)	

**Table 2:** Mean difference, standard deviation (SD) and lower and upper 95% limits of agreement (LoA) for all measures. Green/shaded boxes indicate that measures are within the acceptable limits of agreement, as defined by Pederson et al., 2023 and Brennan et al., 2021 [9,15] (i.e., ±0.05 mm for axial length, ±0.40 D for SER and ±0.06 mm for corneal curvature).

		Mean Difference (SD)	95% Lower LoA	95% Upper LoA
Axial length (mm)	IOL Master 700 v Myopia Master	0.02 (0.02)	−0.02	0.06
	Myopia Master First Examiner v Second Examiner	0.00 (0.02)	−0.04	0.04
SER (D)	Myopia Master v NVisionK 5001	−0.44 (0.24)	−0.91	0.03
	Myopia Master First Examiner v Second Examiner	−0.01 (0.17)	−0.33	0.32
Corneal curvature (mm)	IOL Master 700 v Myopia Master	0.025 (0.03)	−0.03	0.08
	Myopia Master First Examiner v Second Examiner	0.01 (0.02)	−0.03	0.05

## Data Availability

The raw data supporting the conclusions in this article will be made available by the authors on request. This article is a revised and expanded version of an abstract presented at ARVO in 2023 [31].

## Abbreviations

The following abbreviations are used in this manuscript:

WCO	World Council of Optometry
IMI	International Myopia Institute
SS-OCT	Swept-source ocular coherence tomography
ETDRS	Early Treatment of Diabetic Retinopathy Study
SER	Spherical equivalent refraction
MD	Mean deviation
SD	Standard deviation
LoA	Limits of agreement
Orthok	Orthokeratology
